# Association of personalized and tumor-informed ctDNA with patient survival outcomes in pancreatic adenocarcinoma

**DOI:** 10.1093/oncolo/oyae155

**Published:** 2024-07-17

**Authors:** Gregory P Botta, Maen Abdelrahim, Ronald L Drengler, Vasily N Aushev, Abdullah Esmail, George Laliotis, Chris M Brewer, Giby V George, Steven M Abbate, Sreenivasa R Chandana, Mohamedtaki A Tejani, Midhun Malla, Dhruv Bansal, Samuel Rivero-Hinojosa, Erik Spickard, Nicole McCormick, Michael Cecchini, Jill Lacy, Naomi Fei, Pashtoon Murtaza Kasi, Anup Kasi, Farshid Dayyani, Diana L Hanna, Shruti Sharma, Meenakshi Malhotra, Alexey Aleshin, Minetta C Liu, Adham Jurdi

**Affiliations:** Division of Hematology and Oncology, Department of Medicine, University of California San Diego Moores Cancer Center, La Jolla, CA 92037, United States; Section of GI Oncology, Department of Medical Oncology, Houston Methodist Cancer Center, Houston, TX 77030, United States; Medical Oncology, The START Center for Cancer Care, San Antonio, TX 78229, United States; Oncology, Natera, Inc., Austin, TX 78753, United States; Section of GI Oncology, Department of Medical Oncology, Houston Methodist Cancer Center, Houston, TX 77030, United States; Oncology, Natera, Inc., Austin, TX 78753, United States; Oncology, Natera, Inc., Austin, TX 78753, United States; Oncology, Natera, Inc., Austin, TX 78753, United States; Medical Oncology, The START Center for Cancer Care, San Antonio, TX 78229, United States; Department of Gastrointestinal Medical Oncology, Cancer & Hematology Centers of Western Michigan, Grand Rapids, MI 49546, United States; Hematology and Oncology, Advent Health Cancer Institute, Orlando, FL 32804, United States; Section of Hematology and Oncology, University of Alabama at Birmingham, AL 35233,United States; Department of Hematology-Oncology, Saint Luke’s Cancer Institute, Kansas City, MO 64111, United States; Oncology, Natera, Inc., Austin, TX 78753, United States; Oncology, Natera, Inc., Austin, TX 78753, United States; Oncology, Natera, Inc., Austin, TX 78753, United States; Department of Internal Medicine (Medical Oncology), Yale University School of Medicine, New Haven, CT 06520, United States; Department of Internal Medicine (Medical Oncology), Yale University School of Medicine, New Haven, CT 06520, United States; Division of Hematology, Oncology and Blood and Marrow Transplantation, University of Iowa Hospitals and Clinics, Iowa City, IA 52242, United States; Division of Internal Medicine, Department of Oncology/Hematology, Weill Cornell Medicine, New York, NY 10021, United States; Division of Medical Oncology, Department of Medicine, Kansas University Cancer Center, Kansas City, KS 66160, United States; Chao Family Comprehensive Cancer Center, University of California Irvine, Orange, CA 92868, United States; Division of Medical Oncology, Norris Cancer Center, Keck Medicine of USC, Los Angeles, CA 90033, United States; Oncology, Natera, Inc., Austin, TX 78753, United States; Oncology, Natera, Inc., Austin, TX 78753, United States; Oncology, Natera, Inc., Austin, TX 78753, United States; Oncology, Natera, Inc., Austin, TX 78753, United States; Oncology, Natera, Inc., Austin, TX 78753, United States

**Keywords:** ctDNA, molecular residual disease, pancreatic adenocarcinoma, KRAS

## Abstract

**Introduction:**

Personalized and tumor-informed circulating tumor DNA (ctDNA) testing is feasible and allows for molecular residual disease (MRD) identification in patients with pancreatic ductal adenocarcinoma (PDAC).

**Methods:**

In this retrospective analysis of commercial cases from multiple US institutions, personalized, tumor-informed, whole-exome sequenced, and germline-controlled ctDNA levels were quantified and analyzed in patients with PDAC. Plasma samples (*n* = 1329) from 298 clinically validated patients were collected at diagnosis, perioperatively (MRD-window; within 2-12 weeks after surgery, before therapy), and during surveillance (>12 weeks post-surgery if no ACT or starting 4 weeks post-ACT) from November 2019 to March 2023.

**Results:**

Of the initially diagnosed patients with stages I-III PDAC who went for resection, the median follow-up time from surgery was 13 months (range 0.1-214). Positive ctDNA detection rates were 29% (29/100) and 29.6% (45/152) during the MRD and surveillance windows, respectively. Positive ctDNA detection was significantly associated with shorter DFS within the MRD window (median DFS of 6.37 months for ctDNA-positive vs 33.31 months for ctDNA-negative patients; HR: 5.45, *P* < .0001) as well as during the surveillance period (median DFS: 11.40 months for ctDNA-positive vs NR for ctDNA-negative; HR: 12.38, *P* < .0001). Additionally, DFS was significantly better with *KRAS* wildtype status followed by *KRAS*^*G12R*^ (HR: 0.99, *P* = .97), *KRAS*^*G12D*^ (HR: 1.42, *P* = .194), and worse with *KRAS*^*G12V*^ (HR: 2.19, *P* = .002) status. In multivariate analysis, ctDNA detection at surveillance was found to be the most significant prognostic factor for recurrence (HR: 24.28, *P* < .001).

**Conclusions:**

Perioperative tumor-informed ctDNA detection in PDAC is feasible across all stages and is associated with patient survival outcomes.

## Teaser

Personalized, tumor-informed circulating tumor DNA (ctDNA) has been established as a biomarker for molecular residual disease across several tumor types. This study analyzed ctDNA levels in plasma samples from patients with initial stages I-III pancreatic adenocarcinoma who were potential surgical candidates.

**Implications for practice**

Personalized, tumor-informed circulating tumor DNA (ctDNA) has been established as a biomarker for molecular residual disease (MRD) across several tumor types. Here we analyzed ctDNA levels in plasma samples from patients with initial stages I-III pancreatic adenocarcinoma (PDAC) who were potential surgical candidates. Samples were collected at diagnosis, perioperatively (MRD-window; within 2-12 weeks after surgery, prior to therapy) and during surveillance (>12 weeks post-surgery if no ACT or starting 4 weeks post-ACT). Positive ctDNA detection was found to be significantly associated with shorter disease-free survival within the MRD window and during the surveillance period as was the specific *KRAS* G12V mutation. Furthermore, in multivariate analysis, ctDNA detection at any time post-operatively was found to be the most significant prognostic factor for recurrence, not CA19-9. Taken together, our data highlights the feasibility of perioperative tumor-informed ctDNA analysis in PDAC across all stages, its utility in risk stratification and prediction of disease recurrence, and its association with patient survival outcomes.

## Introduction

Pancreatic cancer accounts for 3% of all cancer diagnoses and nearly 7% of all cancer-related deaths.^1^ It is predicted to become the second leading cause of cancer-related death by the year 2030.^2^ Approximately 80% of patients are diagnosed with locally advanced unresectable or metastatic disease, such that only 15%-20% of patients with pancreatic ductal adenocarcinomas (PDACs) are fit to be surgically resected at diagnosis.^3-5^ Even for patients that undergo potentially curative resection, approximately 75% recur systemically after curative-intent surgery and adjuvant chemotherapy.^6-8^ The reported overall 5-year survival rate of PDAC for any stage is 12% and for those with metastatic disease is only 3%.^9^

In the curative setting for patients with resectable PDAC, National Comprehensive Cancer Network (NCCN) guidelines recommend 6 months of adjuvant chemotherapy (ACT) after resection to eradicate micrometastatic disease.^10^ However, the NCCN panel and many expert pancreatic cancer centers now recommend peri-operative or total neoadjuvant chemotherapy (NAC) for a total of 6 months in patients who are at high-risk of early micrometastatic spread but still considered for surgical resection. These higher-risk patients have tumor-related symptoms, elevated cancer antigen 19-9 (CA19-9), large primary tumors, bulky regional nodes, and imaging findings concerning vascular involvement. American Society of Clinical Oncology (ASCO) guidelines recommend NAC for patients with resectable PDAC who cannot undergo upfront surgery.^11^

Though clinical trials have shown the benefit of ACT over resection alone in improving overall survival (OS) regardless of any pathological N stage and margin status,^12,13^ it is crucial to refine the treatment paradigm with tools that can help stratify high-risk patients who are most likely to experience disease recurrence/progression and may most benefit from neoadjuvant and adjuvant systemic therapy as well as clinical trials. Currently, clinical symptoms, serum biomarker CA 19-9 (sialylated Lewis A blood group antigen), along with contrast-enhanced imaging in the form of CT or MRI, are used for disease surveillance.^10^ However, CA 19-9 is a non-specific biomarker, as it may be elevated in both malignant and benign conditions such as biliary inflammation or obstruction.^14-16^ Additionally, Lewis antigen-negative individuals may be non-secretors of CA 19-9.^16^ Finally, standard imaging modalities rely on the conspicuous detection of a measurable lesion, with exceptional difficulty in determining peritoneal disease, and cannot detect subclinical molecular residual disease (MRD).

Circulating tumor DNA (ctDNA) has emerged as a minimally invasive biomarker that can detect disease recurrence at a molecular level, months ahead of radiological findings or traditional blood biomarkers. Many studies across several cancer types have demonstrated the utility of ctDNA in detecting MRD and guiding treatment decisions.^17-20^ In this study, we investigated the clinical utility of longitudinal ctDNA quantification using a personalized and tumor-informed multiplex (m)PCR next-generation sequencing (NGS) ctDNA assay (Signatera) for MRD detection, monitoring treatment efficacy in the perioperative setting, and predicting recurrence during surveillance. We provide evidence that ctDNA status is prognostic of recurrence and may be used for improved patient risk stratification during peri-operative therapy.

## Methods

### Study cohort and sample collection

**Figure 1.**
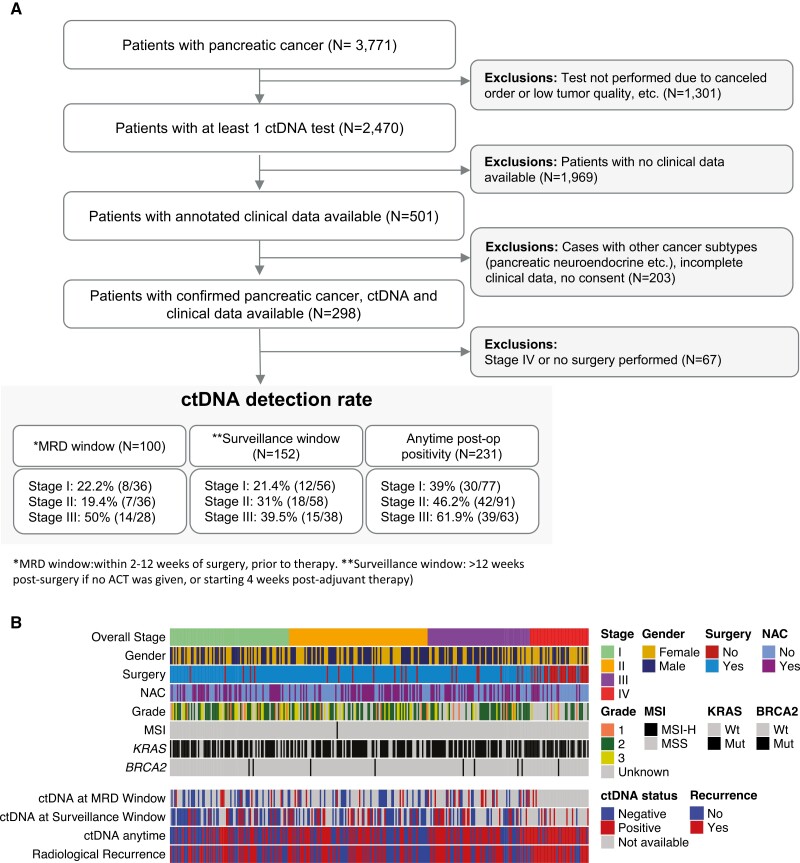
A. Consolidated standards of reporting trials (CONSORT) diagram illustrating patient inclusion and exclusion criteria for sub-analyses. B. Demographics heat map illustrating clinicopathologic features, most frequently observed genetic mutations, ctDNA detection in different settings and overall recurrence rate in this cohort.

In this retrospective study of real-world data in patients with pancreatic cancer from over 10 institutions, data from commercial ctDNA testing collected from November 2019 to March 2023 were analyzed. The overall cohort of 3771 patients was identified from ctDNA requisition forms and histology was confirmed by individual pathology reports. At least one ctDNA result was available for 2470 (65.5%) patients after diagnosis. All samples underwent ctDNA testing using a clinically validated, personalized, tumor-informed ctDNA assay. Only pancreatic adenocarcinoma patients who had physician-validated, clinical data were included in the outcomes analysis. This ensured that the final cohort included only data that was certified by the treating provider and was void of transposition and abstraction errors inherent in third-party electronic medical record (EMR) data exchange. Inclusion criteria included: patients with confirmed PDAC that had longitudinal ctDNA and DFS data available (*N* = 298) for analyses. Exclusion criteria included: patients with no ctDNA results, no or incomplete validated clinical data or follow-up, other histologic subtypes besides ductal adenocarcinoma (pancreatic neuroendocrine tumors, etc.), or no informed consent (*N* = 203). Patients who had no surgery data available or had stage IV disease were excluded (*N* = 67) from the final survival analysis as they rarely undergo curative-intent surgery. Decision to proceed to surgery was determined by each individual center and the patient’s specific treatment team. As such, 231 patients with stages I-III disease were included in the survival analysis. ctDNA analysis was performed on plasma samples collected pre- and postoperatively (MRD-window; within 2-12 weeks of surgery, prior to therapy) and during surveillance (>12 weeks post-surgery, if no ACT was given, or starting 4 weeks post-ACT). Twelve weeks was selected as the cutoff for MRD and surveillance windows from precedent set in previous pancreatic cancer clinical trials^21,22^ (Figure 1A).

Clinicopathologic information was collected for all patients (Supplementary Figure S1A). All patients received treatment and follow-up at the discretion of the treating physician. The ctDNA measurements were conducted by Natera laboratory personnel who were blinded to clinical data including disease-free survival (DFS) and overall survival. The ctDNA statistical analysis plan was developed prior to unblinding the clinical data. Data were de-identified prior to analysis. Retrospective analysis of de-identified data, including ctDNA results and clinicopathologic factors collected for quality assurance purposes under 45 CFR 164.501 was determined to be exempt research by an independent Institutional Review Board—Salus #20099-04 through approved protocol# 20-049-ALL.

#### Biospecimen collection and processing

Tumor DNA was extracted from formalin-fixed and paraffin-embedded (FFPE) tissue from biopsies or resected tumor with sufficient cellularity for all patients with pancreatic cancer. For the germline DNA analysis, a single blood sample was collected in a 6 mL EDTA test tube. Blood samples for ctDNA analyses were collected in 2, 10 mL Streck tubes throughout the patients’ ACT/surveillance course.

### Personalized mPCR-based NGS assay for ctDNA detection

Briefly, a clinically validated, personalized, tumor-informed, 16-plex mPCR NGST assay (Signatera^TM^) was used for the detection and quantification of ctDNA, as previously published.^20^ Briefly, FFPE tumor blocks and matched normal DNA blood samples were whole exome sequenced (WES). The matched normal samples were used to remove germline mutations and alterations related to clonal hematopoiesis of indeterminate potential. Based on the results of WES, 16 patient-specific, somatic, single-nucleotide variants were selected for each patient. Cell-free DNA was extracted from a median of 9.7 mL of plasma (range: 2.1-11.5 mL). Universal libraries were created by end repair, A-tailing, and ligation with custom adapters. Next, libraries were amplified by mPCR, barcoded, pooled, and sequenced on an NGS platform. Plasma samples with at least 2 variants detected were defined as ctDNA-positive, and ctDNA concentration was reported in mean tumor molecules (MTM)/mL of plasma.

### Statistical analysis

The primary outcome measured was DFS, as determined by radiological findings and validated clinical documentation by the treating physician. DFS was measured from the date of surgery to the first documented sign of radiological recurrence, either locoregional or distant, or death from any cause and was censored at the last follow-up or death. Survival analysis was performed using the Kaplan–Meier method (R version 4.1). For visualization, data were censored at 60 months post-surgery due to a lack of events after 60 months of follow-up. A multivariable Cox proportional hazards model was used to assess the most significant prognostic factor associated with DFS. All *P*-values were based on 2-sided testing; differences were considered significant at *P* ≤ .05.

## Results

### Patient cohort

A total of 1329 plasma samples were collected from 298 clinically validated patients considered for surgery. The majority of the patients tested had resectable/borderline resectable tumors as defined by the treating physician (*N* = 258; 86%). The patients with PDAC were defined as stage I (*N* = 85; 28%), stage II (*N* = 99; 33%), stage III (*N* = 73; 24%), and stage IV (41; 14%) (Supplementary Figure S1A). Surgery was performed on 83% (*N* = 248) of the patients, with 47% (*N* = 140) receiving neoadjuvant treatment. The median age of the cohort was 67.1 years (range: 28.2-88.4 years). Detailed patient demographics are available in Supplementary Figure S1A and 1B. On analyzing the timing of the first ctDNA time point tested <6 months post-surgery, 68.9% (100/145) were tested first during the MRD window (2-4 weeks: 21.38% [31/145], 4-8 weeks: 40% [58/145], 8-12 weeks: 14.48% [21/145]) and 21.38% (31/145) were tested first during the surveillance window (Supplementary Figure 1B).

For patients included in the survival analysis (*N* = 231), the median time of follow-up from surgery was 13 months (0.1-107 months). The positive ctDNA detection rate was 29% (29/100) and 29.6% (45/152) during the MRD and surveillance windows, respectively (Figure 1A). On examining the correlation of ctDNA detection rate with disease stage during the surveillance window, we observed any time postoperative ctDNA-positivity (*N* = 231) increased with stage: 39% (30/77) for stage I, 46.2% (42/91) for stage II, and 61.9% (39/63) for stage III (Figure 1A). Additionally, patients with stage III disease had a higher rate of radiologic recurrence 44.7% (17/38) than patients with stage I (32.1%; 18/56) or stage II (36.2%; 21/58).

### Association of ctDNA detection during MRD window with patient outcomes

**Figure 2.**
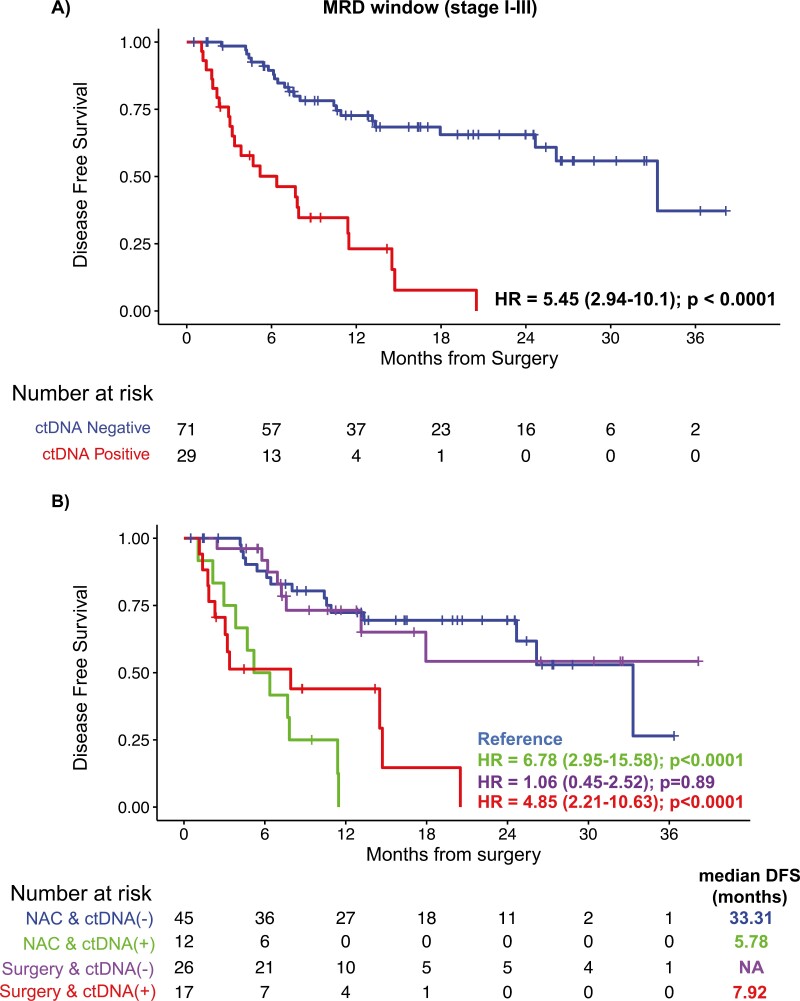
ctDNA-based MRD testing is predictive of survival outcomes in postsurgical patients with pancreatic cancer. A. Kaplan-Meier estimates for DFS stratified by ctDNA-negative and ctDNA-positive status from 2 to 12 weeks after surgery. B. Kaplan-Meier estimates for DFS stratified by ctDNA-negative and ctDNA-positive status at MRD in patients who either received neoadjuvant therapy or up-front surgery. HRs and 95% CIs were calculated using the Cox proportional hazard model. *P* values were calculated using the 2-sided log-rank test.

For survival analysis only stages I-III (*N* = 100) clinically validated patients with ctDNA and DFS data available within the MRD window were included. ctDNA-positivity was associated with a significantly shorter median DFS (mDFS of 6.37 months for ctDNA-positive vs 33.31 months for ctDNA-negative patients; HR: 5.45, 95% CI, 2.94-10.1, *P* < .0001) (Figure 2A) and this trend was observed across all stages (stage I: HR 18.64; *P* < .0001, stage II: HR 7.92; *P* = .0003, stage III: HR 8.61; *P* = .007) (Supplementary Figure S2A–C). Among patients analyzed during the MRD window (2-12 weeks after surgery), 57% (57/100) received NAC and 43% (43/100) received upfront surgery, with a positive ctDNA detection rate of 21.05% (12/57) and 39.53% (17/43), respectively. Compared to ctDNA-negative patients who received NAC, ctDNA-negative patients receiving upfront surgery had similar outcomes (HR 1.06, 95% CI, 0.45-2.52, *P* = 0.89), while patients who remained ctDNA-positive after NAC or upfront surgery had a higher rate of recurrence (RR) (RR for ctDNA-positive with NAC: 91.6%, 11/12; ctDNA-positive with surgery: 70.5%, 12/17 vs ctDNA-negative with NAC: 33.3%%, 15/45; ctDNA-negative with surgery: 30.8%, 8/26) and demonstrated significantly inferior DFS; HR 6.78, 95% CI, 2.95-15.58, *P* < .0001 and, HR 4.85, 95% CI, 2.21-10.63; *P* < .0001, respectively (Figure 2B).

### Benefit of ACT in patients receiving neoadjuvant treatment vs upfront surgery, stratified by ctDNA status

**Figure 3.**
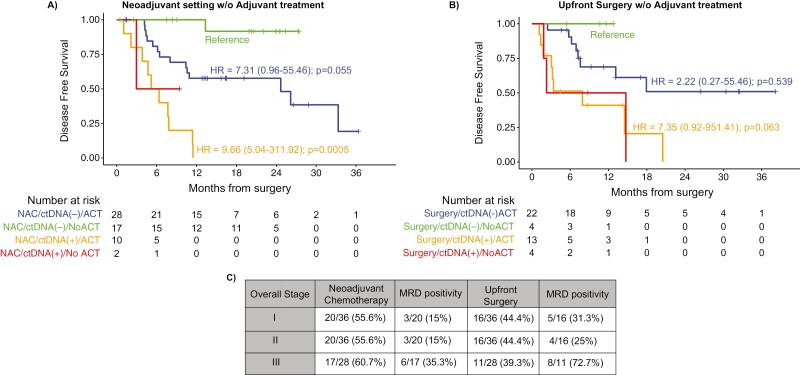
ctDNA-based MRD testing is predictive of survival outcomes in postsurgical patients with pancreatic cancer. A. Kaplan-Meier estimates for DFS stratified by ctDNA-negative and ctDNA-positive status in patients who received neoadjuvant therapy with or without adjuvant therapy. B. Kaplan-Meier estimates for DFS stratified by ctDNA-negative and ctDNA-positive status in patients who received upfront surgery with or without adjuvant therapy. HRs and 95% CIs were calculated using the Cox proportional hazard model. *P* values were calculated using the 2-sided log-rank test. C. Stage-wise MRD positivity rate in patients receiving neoadjuvant therapy vs upfront surgery.

In an exploratory analysis, we further investigated the benefit of ACT in patients who received NAC versus upfront surgery, stratified by post-surgical MRD ctDNA status. In general, ACT for 6 months is the standard of care in patients who undergo upfront surgery for resectable PDAC. In our cohort, we observed a limited number of patients in the upfront surgery cohort who did not receive ACT, in line with other adjuvant studies and more than in perioperative studies.^21-23^ In patients who receive NAC, the value of ACT after surgery is not well established as there is no data yet showing changes in survival if ACT is omitted or not.^22^ In our study, when stratified by ctDNA status, patients who received NAC with ctDNA-positivity in the MRD window showed worse outcomes regardless of receiving ACT (Figure 3). This suggests that patients who are ctDNA-positive after NAC and surgery may likely have chemo-resistant disease and should be considered for a different ACT regimen (“switch therapy”) or enrollment into clinical trials. Our findings are limited by the non-randomized nature of this study with patients presenting with more advanced disease being more likely to receive NAC. Unexpectedly, if a patient was ctDNA negative after completing neoadjuvant chemotherapy or surgery, their DFS trended down if they received ACT vs those who did not.

#### Association of ctDNA detection during surveillance with patient outcomes

**Figure 4.**
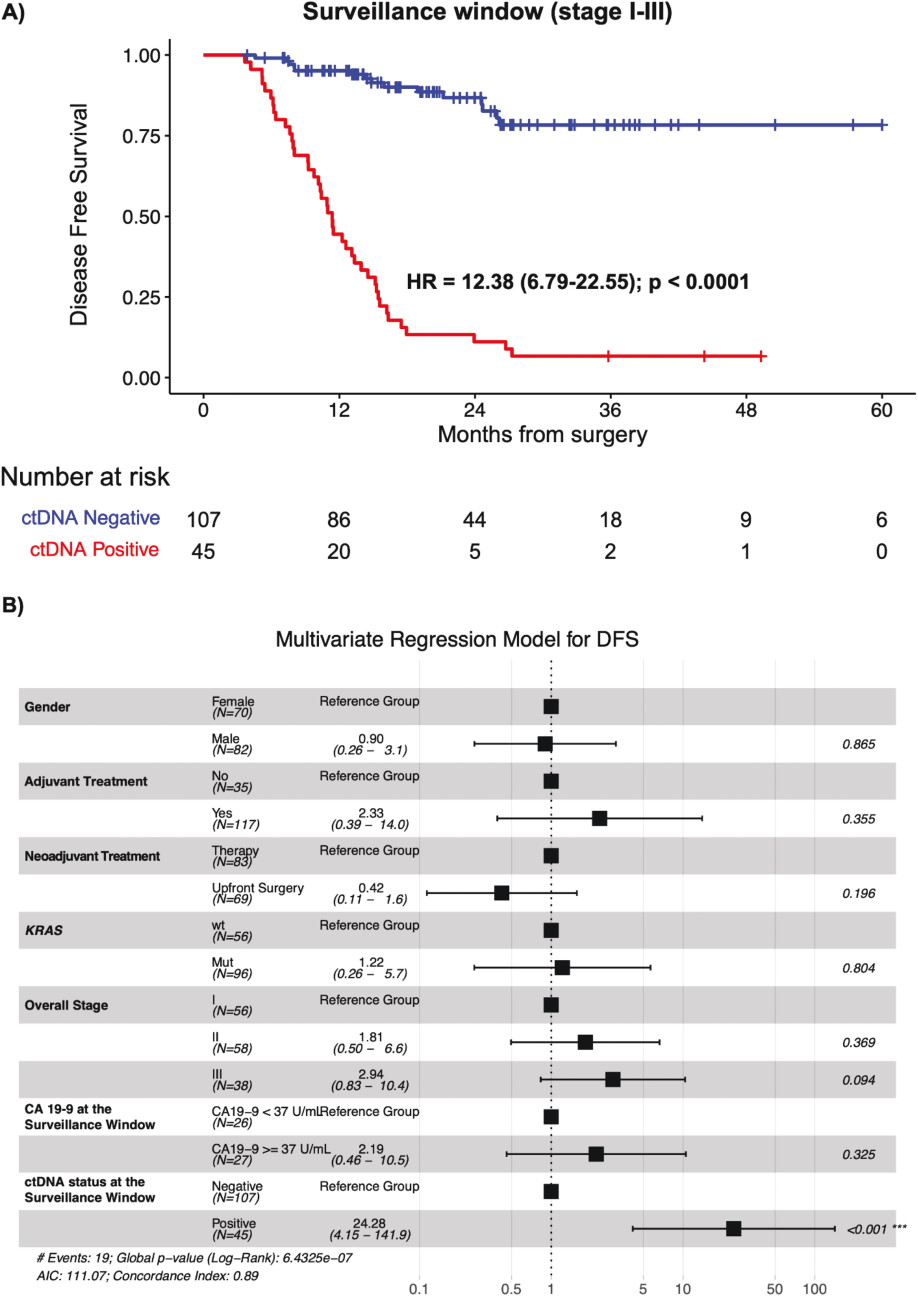
ctDNA-based testing during the surveillance window is predictive of survival outcomes in postsurgical patients with pancreatic cancer. A. Kaplan-Meier estimates for DFS stratified by ctDNA-negative and ctDNA-positive status from >12 weeks after surgery. HRs and 95% CIs were calculated using the Cox proportional hazard model. *P* values were calculated using the 2-sided log-rank test. B. Forest plot depicting the multivariate analysis for recurrence in patients with stages I-III pancreatic cancer. Various prognostic factors and their association with DFS, as indicated by HR, were analyzed across the cohort using the 2-sided Wald chi-squared test. The unadjusted HRs (squares) and 95% CIs (horizontal lines) are shown for each prognostic factor. Vertical dotted line, the null hypothesis.

Similarly, within the surveillance period (*N* = 152), ctDNA-positivity was strongly associated with reduced median DFS (mDFS: 11.4 months for ctDNA-positive vs NR for ctDNA-negative; HR: 12.38, 95% CI, 6.79-22.55, *P* < .0001) (Figure 4A). This trend remained consistent and significant across all stages (stage I: HR 11; *P* < .0001, stage II: HR 9.76; *P* < .0001, stage III: HR 21.54; *P* < .0001) (Supplementary Figure 2D-F). To examine the relative contribution of ctDNA positivity to recurrence risk, we conducted a multivariate analysis with available prognostic factors (gender, NAC, ACT, *KRAS* mutation, stage, and CA19-9) in this cohort. Only ctDNA detection during the surveillance window was found to be an independent and significant predictor of DFS (HR: 24.28, 95% CI, 4.15-141.9, *P* < .001), not CA19-9 level, *KRAS* mutational status, gender, stage, or peri-operative treatment. Even when accounting for concentration differences in the standard of care biomarker CA 19-9 (less than or equal to 37 U/mL), there was no significance in its ability to predict DFS in this cohort (*P* = .325) (Figure 4B).

#### Association of ctDNA dynamics with patient outcomes

**Figure 5.**
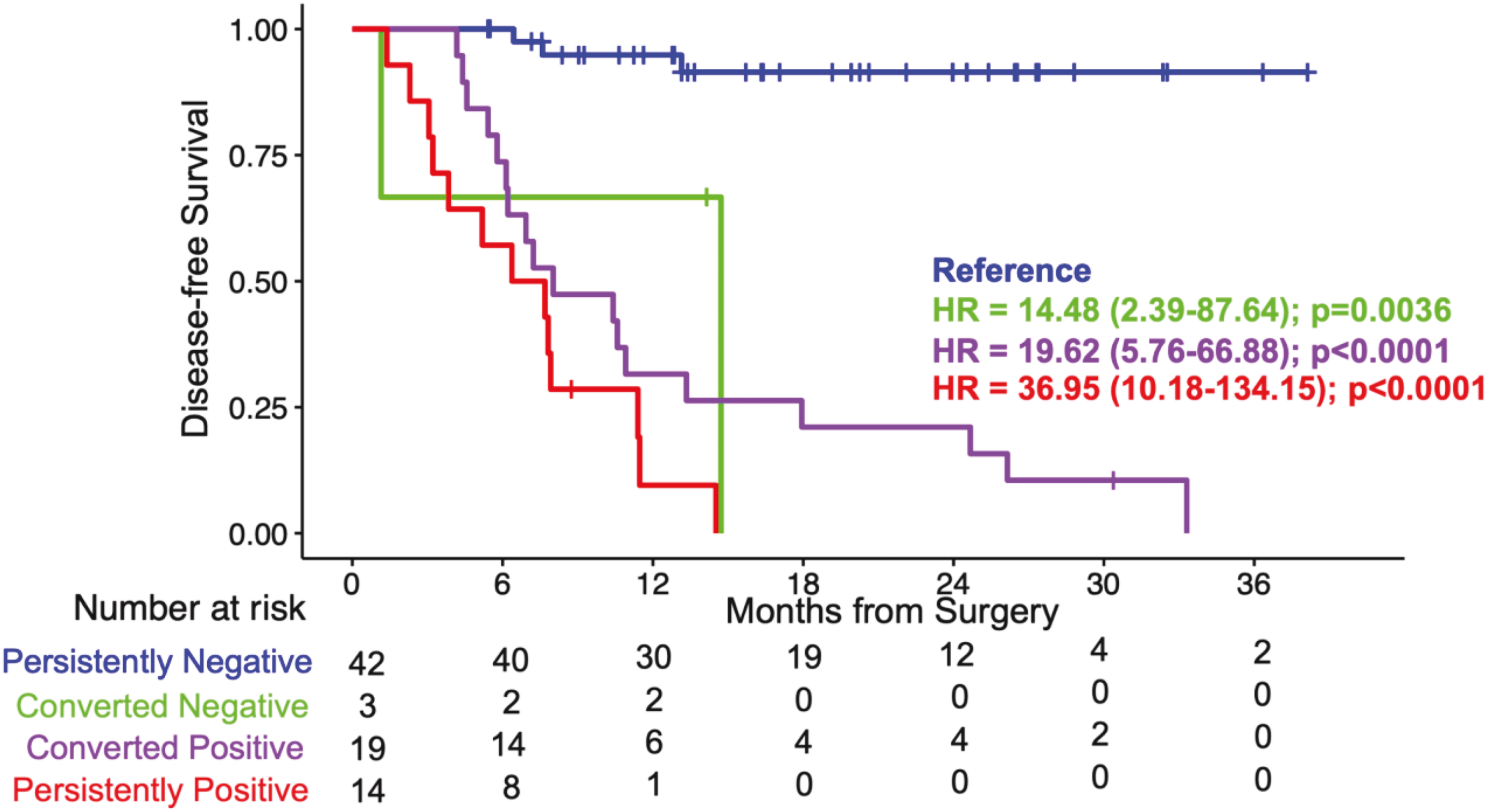
ctDNA dynamics with patient outcomes. Kaplan-Meier estimates for DFS according to ctDNA dynamics in patients that had post-surgical MRD time point to any time during surveillance in patients receiving adjuvant chemotherapy or the first subsequent surveillance timepoint in patients with no adjuvant chemotherapy. HRs and 95% CIs were calculated using the Cox proportional hazard model. *P* values were calculated using the 2-sided log-rank test.

Next, we investigated whether tumor-informed ctDNA dynamics postoperatively correlate with DFS. We compared ctDNA status in patients at the post-surgical MRD time point to anytime during surveillance as defined in Methods. Out of a total of 78 patients included in this analysis, 15.85% (14/78) remained ctDNA-positive and 58.53% (42/78) remained ctDNA-negative, whereas 23.17% (19/78) converted from negative to positive and 2.43% (3/78) converted from positive to negative (Figure 5). On comparing to the RR of patients who were persistently negative (3.3%, 3/42), a significantly higher RR was observed for patients who either remained persistently positive (RR: 92.9% (13/14); HR 36.95, 95% CI, 10.18-134.15; *P* < .0001) or converted from negative to positive (RR: 94.7% (18/19); HR 19.62, 95% CI, 5.76-66.88; *P* < 0.0001) (Figure 5).

### Patient-level genomic characteristics

**Figure 6.**
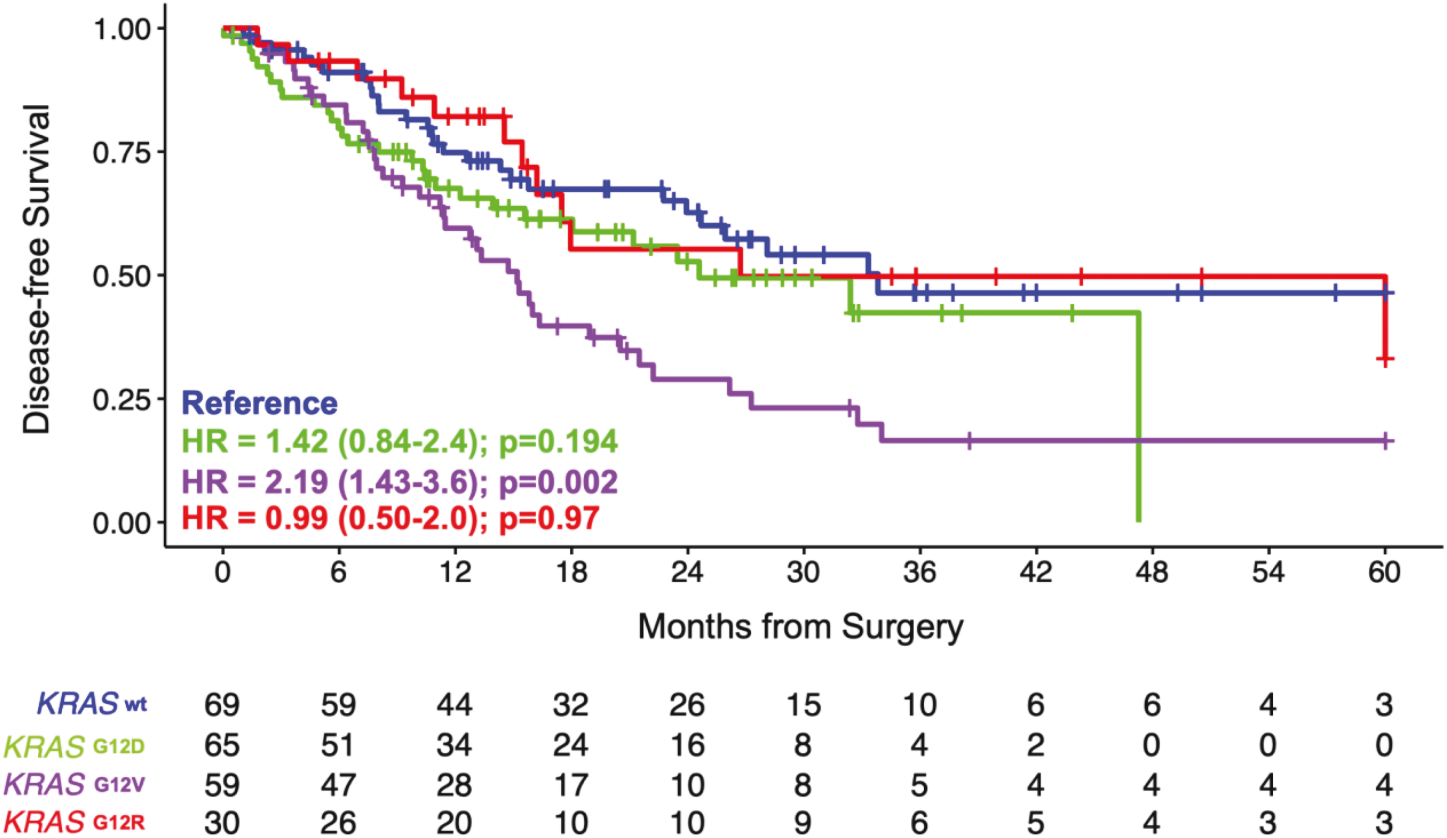
KRAS G12V and G12D were associated with worse DFS. Kaplan-Meier estimates for DFS stratified by KRAS wild-type and mutations (G12D, G12V, and G12). HRs and 95% CIs were calculated using the Cox proportional hazard model. *P* values were calculated using the 2-sided log-rank test.

We performed an exploratory analysis on the WES data available from Natera’s commercial database to identify genomic profiles and characteristics for all patients (*N* = 298). WES results revealed mutant *KRAS* (72%) and loss of *BRCA 1/2* (10%) to be the most commonly mutated genes observed when considering non-synonymous variants. There were 28% (84/298) KRAS wild-type patients in our analysis. Of the *KRAS* mutations (*n* = 215), 37.7% (81/215) were G12D, 33.9% (73/215) were G12V, and 18.6% (40/215) were G12R, with no G12C mutations found in this cohort. No significant association was observed between MSI status 0.3% (1/298) and stage of disease (Supplementary Figure 1A). No trends were observed between tumor stage and frequency of any genetic mutation. In our cohort, *KRAS* G12V and G12D were associated with significantly worse DFS when compared to KRAS wildtype (Figure 6).

## Discussion

Although the ctDNA detection rate has historically been low in patients with PDAC (attributable to the unique tumor biology, paucity of biopsy samples due to fine needle aspiration (FNA) technique, and high content of extracellular matrix causing overall low tumor content), sensitive and specific methods for quantification may enable more accurate characterization.^24^ ctDNA as measured by a single *KRAS* point mutation or standard panels of commonly mutated genes have shown limited sensitivity and specificity to detect MRD.^25-27^ As such, in our study, we demonstrate that tumor-informed ctDNA-positivity within the MRD window and during the surveillance period is both feasible and highly prognostic of poor outcomes in PDAC. This suggests that tumor-informed ctDNA may serve as a more specific biomarker than single *KRAS* gene DNA and tumor-agnostic gene panels, thereby allowing an advised and stratified patient-centered treatment approach in the peri-operative window as well as earlier detection of recurrence.^24^

Comparing the tumor-agnostic and tumor-informed ctDNA approaches, Watanabe et al demonstrated an improved ctDNA detection rate for the tumor-informed approach in a resectable PDAC Japanese population at 2 University hospitals (*n* = 145): 39% (28/71) vs 56% (40/71) in treatment-naïve patients and 31% (23/74) vs 36% (27/74) in neoadjuvant-treated patients, respectively. It was also shown in this small cohort that detectable ctDNA was associated with shorter DFS (*P* = .0010).^27^ In our multi-institutional and ethnic dataset, the ctDNA positivity rate was 28.18% in the MRD window, and 29.48% during the surveillance window. In a sub-analysis, the ctDNA-positive detection rate within the MRD window in patients who received NAC followed by surgery vs those who underwent upfront surgery was 21.1% and 39.5%, respectively. More interestingly, the prognostic value of serial ctDNA-based MRD testing in this sub-analysis revealed a recurrence rate of 92.9% and 94.7% for patients who remained persistently positive or converted positive, respectively, suggesting an aggressive disease biology refractory to the chemotherapy selected. We would envision that clinical trials utilizing novel drugs or “switch-therapy” (ie, mFOLFIRINOX to gemcitabine/nab-paclitaxel) may be best used in tumor-informed ctDNA persistently positive PDAC patients given that they are either harboring active disease or are at an extremely high-risk for recurrence. Conversely, our analysis also clearly delineates improved DFS for patients who clear their ctDNA at any time regardless of upfront surgical or NAC treatment. Clinical trials could be developed that evaluate outcomes with a limited number of cycles or reduced dosing of chemotherapy in these ctDNA-negative patients.

Previously, using digital droplet PCR (ddPCR), Hadano et al evaluated only *KRAS* point mutation ctDNA in PDAC patients in the post-surgical setting.^28^ They reported the median OS to be 27.6 months for patients who were mutant *KRAS* ctDNA-negative compared to 13.6 months for those who were mutant *KRAS* ctDNA-positive (*P* < .0001).^28^ More recently, using a tumor-uninformed, agnostic blood-based panel, Patel et al also showed that higher levels of ctDNA (%) were associated with worse OS (HR: 4.35; 95% CI, 1.85-10.24, *P* = .001).^29^ Another study utilizing a tumor-agnostic NGS-based panel reported that PDAC patients with postoperative ctDNA-positive status displayed a significantly reduced DFS compared to those with ctDNA-negative status (HR: 5.20, *P* = .019).^25^

Our data represent the largest real-world, personalized tumor-informed ctDNA data analysis across the pancreatic cancer treatment spectrum and strengthen the prognostic value of ctDNA in both the postsurgical MRD (HR: 5.45, 95% CI, 2.94-10.1, *P* < .0001) and the surveillance setting (HR: 12.38, 95% CI, 6.79-22.55, *P* < .0001) in this disease. Further, whole exome sequencing of each patient will permit subsequent studies evaluating gene signatures of clinical responders and possible prediction of appropriate therapy regimens in this cohort.

Presently, CA 19-9 is the standard antigen biomarker used for the detection and surveillance of PDAC. However, its limitations include low sensitivity (with a false positive rate of 47%, especially in the presence of endobiliary stents) and lack of uniform secretion in the population (as 5%-10% of individuals are incapable of producing CA 19-9).^24,30^ In our study, we found that elevated CA 19-9 along with other standard clinicopathological features were not correlated with DFS in PDAC patients (*P* = .325). Moreover, in a multivariate analysis, ctDNA positivity correlated with patient survival outcomes more strongly than CA19-9 or any other clinicopathological feature, suggesting that ctDNA may prove a promising biomarker for the detection of pancreatic cancer MRD, assessment of therapeutic response, and early identification of disease recurrence with a higher sensitivity and specificity than traditional antigen biomarkers.

Our study possesses several limitations, including patient and plasma timepoint heterogeneity and the use of FNA that impacted the procurement of sufficient tumor tissue. We also acknowledge that there may have been an inherent selection bias given the retrospective, pragmatic nature of this investigation. However, this may have been partly accounted for by our larger cohort of samples. While we observed a clinically significant lead time (mean: 101 days; range: 1-421 days), this study uniquely presents the clinical utility of a tumor-informed ctDNA assay, wherein some treating physicians may have altered their surveillance regimen based on ctDNA results, with a positive ctDNA result triggering an earlier imaging study, thereby artificially shortening the observed lead time. Of note, we did observe that a majority (40%) of the providers ordered the first post-operative ctDNA tests for their patients between 4 and 8 weeks after surgery, relevant to adjuvant treatment decision-making. Since timing of ctDNA testing is crucial and may impact detection rates, previous studies have demonstrated that waiting at least 2 weeks after surgical resection is necessary to reduce surgery-induced increased cell-free DNA levels, which may artificially attenuate the ctDNA detection rate.^31,32^

Currently, several trials are utilizing ctDNA for treatment stratification as well as evaluating whether ctDNA dynamics may serve as a surrogate endpoint for treatment efficacy, across solid tumors.^33^ For example, the multicenter ELYMIN18.2 CAR-T trial of pancreatic and patients with gastric cancer that tumor-informed ctDNA correlates with response to CLDN18.2 CAR-T-cell therapy. In this phase I study, OS was higher (9.1 months vs 3.7 months) in those who achieved anytime undetectable ctDNA.^34^ These trials are “first-movers” into the utilization of tumor-informed ctDNA in pancreatic cancer clinical trial design and will validate what the prognostic outcomes of positive and negative ctDNA are for these unique patient populations.

We present the largest cohort of perioperative, clinically validated patients with pancreatic cancer with longitudinal tumor-informed, personalized ctDNA results (*n* = 298). Overall, ctDNA positivity for MRD predicts a significantly shorter DFS whether this ctDNA positivity is after NAC or surgery. Further, it does not appear that adjuvant chemotherapy after NAC or surgery reduces DFS if ctDNA is persistently positive after these therapeutic interventions. The unexpected trend that pancreatic cancer patients with negative tumor-informed ctDNA after neoadjuvant therapy or surgery and who completed adjuvant therapy had decreased DFS needs to be taken cautiously given the exploratory nature of this analysis. There is a possibility that the patients who did not receive adjuvant therapy had exceptional responses and those who received adjuvant therapy were those with high-risk pathologic features. However, an opportunity to evaluate the perioperative SWOG 1505 trial data outcomes between pancreatic cancer patients who completed both NAC and surgery with and without adjuvant treatment will assist in determining the validity of this observation.^22,35^ Patients past the treatment window and on surveillance with tumor-informed ctDNA positivity also had a reduced DFS and in multivariate analysis, ctDNA was the only significant prognostic variable in this cohort; not CA 19-9. Dynamically, patients who converted from negative to positive over the course of their surveillance had significantly worse DFS than those who converted to or were persistently ctDNA negative, providing another potential “high-risk” or “treatment-failure” patient cohort to enroll in an interventional clinical trial. In our cohort, a total of 111 patients experienced radiological relapse, of whom 27 had a local relapse. All 27 patients had ctDNA time points available and of these 74.07% (20/27) were ctDNA-positive at any time post-surgery, prior to their local relapse. This underscores that tumor-informed ctDNA is capable of detecting local relapse within the peritoneum at a time when imaging may not be clear.

The implementation of tumor-informed ctDNA into clinical practice will require site protocols to obtain diagnostic tissue volume with high enough cellularity for NGS immediately. This step will characterize patients straightaway and help stratify treatment and prognostic groups. Based on our data, patients with pancreatic cancer who have completed perioperative chemotherapy regimens and who have persistently positive ctDNA will recur over 90% of the time. These patients could be considered at “extremely high risk” of recurrence, but more appropriately should be termed “treatment refractory” and with active disease. Such patients could be identified earlier than imaging progression by tumor-informed ctDNA and placed into novel therapy trials immediately or have their chemotherapy backbone switched to evaluate if their outcomes can be improved. As they continue to have disease within the MRD window, they would be ideal for evaluating other mechanisms of anti-cancer therapy such as vaccines or cellular therapy to see if ctDNA can be converted negative. We should be prudent, however, in immediately initiating systemic chemotherapy in the surveillance window for ctDNA-positive patients without radiographic evidence of disease. There may exist an oligometastatic subset of recurrent patients with PDAC who could move to radiation alone instead of systemic therapy or have lung-only metastasis that foretells an improved outcome. Persistently positive pancreatic cancer patients in the surveillance could do well by enrolling in less toxic interventional trials to delay tumor growth and spread. Patients with pancreatic cancer will always be at the highest risk of recurrence among all solid tumors given our currently limited chemotherapy and surgical techniques, and tumor-informed ctDNA can stratify future clinical trials on appropriate management.

## Supplementary material

Supplementary material is available at *The Oncologist* online.

oyae155_suppl_Supplementary_Material

## Data Availability

The authors declare that all relevant data used to conduct the analyses are available within the article. Any additional request will be reviewed within a time frame of 2-3 weeks by corresponding authors to verify whether the request is subject to any intellectual property or confidentiality obligations. The fully documented code for the R statistical computing environment for analyses and the associated de-identified clinical data related to this manuscript are deposited at the github repository and can be accessed at https://github.com/Natera-TMED/RWE_Pancreas.git.
