# Correction to: Association of personalized and tumor-informed ctDNA with patient survival outcomes in pancreatic adenocarcinoma

**DOI:** 10.1093/oncolo/oyae231

**Published:** 2024-08-19

This is a correction to: Gregory P Botta, Maen Abdelrahim, Ronald L Drengler, Vasily N Aushev, Abdullah Esmail, George Laliotis, Chris M Brewer, Giby V George, Steven M Abbate, Sreenivasa R Chandana, Mohamedtaki A Tejani, Midhun Malla, Dhruv Bansal, Samuel Rivero-Hinojosa, Erik Spickard, Nicole McCormick, Michael Cecchini, Jill Lacy, Naomi Fei, Pashtoon Murtaza Kasi, Anup Kasi, Farshid Dayyani, Diana L Hanna, Shruti Sharma, Meenakshi Malhotra, Alexey Aleshin, Minetta C Liu, Adham Jurdi, Association of personalized and tumor-informed ctDNA with patient survival outcomes in pancreatic adenocarcinoma, *The Oncologist*, 2024;, oyae155, https://doi.org/10.1093/oncolo/oyae155

The originally published version of this paper has been corrected.

In the Abstract, the number of clinically validated patients was originally incorrectly stated as 299, instead of 298, in the following sentence:

Plasma samples (*n* = 1329) from 299 clinically validated patients were collected at diagnosis, perioperatively (MRD-window; within 2-12 weeks after surgery, before therapy), and during surveillance (>12 weeks post-surgery if no ACT or starting 4 weeks post-ACT) from November 2019 to March 2023.

The corrected sentence reads as follows:

Plasma samples (*n* = 1329) from 298 clinically validated patients were collected at diagnosis, perioperatively (MRD-window; within 2-12 weeks after surgery, before therapy), and during surveillance (>12 weeks post-surgery if no ACT or starting 4 weeks post-ACT) from November 2019 to March 2023.

Additionally, in Results, in the subsection entitled “Patient-level genomic characteristics”, an error was present in the following sentence, whereby (84/299) should have been given as (84/298):

There were 28% (84/299) KRAS wild-type patients in our analysis.

The corrected sentence reads as follows:

There were 28% (84/298) KRAS wild-type patients in our analysis.

