# Supplementary material for: Association of personalized and tumor-informed ctDNA with patient survival outcomes in pancreatic adenocarcinoma
Source: Oncologist. 2024 Jul 17;29(10):859–69. doi: 10.1093/oncolo/oyae155 (PMC11449101; doi:10.1093/oncolo/oyae155)
Supplement: oyae155_suppl_Supplementary_Material [file oyae155_suppl_supplementary_material.docx]

**Supplementary Figures for:**

**Association of personalized and tumor-informed ctDNA with patient survival outcomes in pancreatic adenocarcinoma**

Gregory P. Botta et al.

**A**


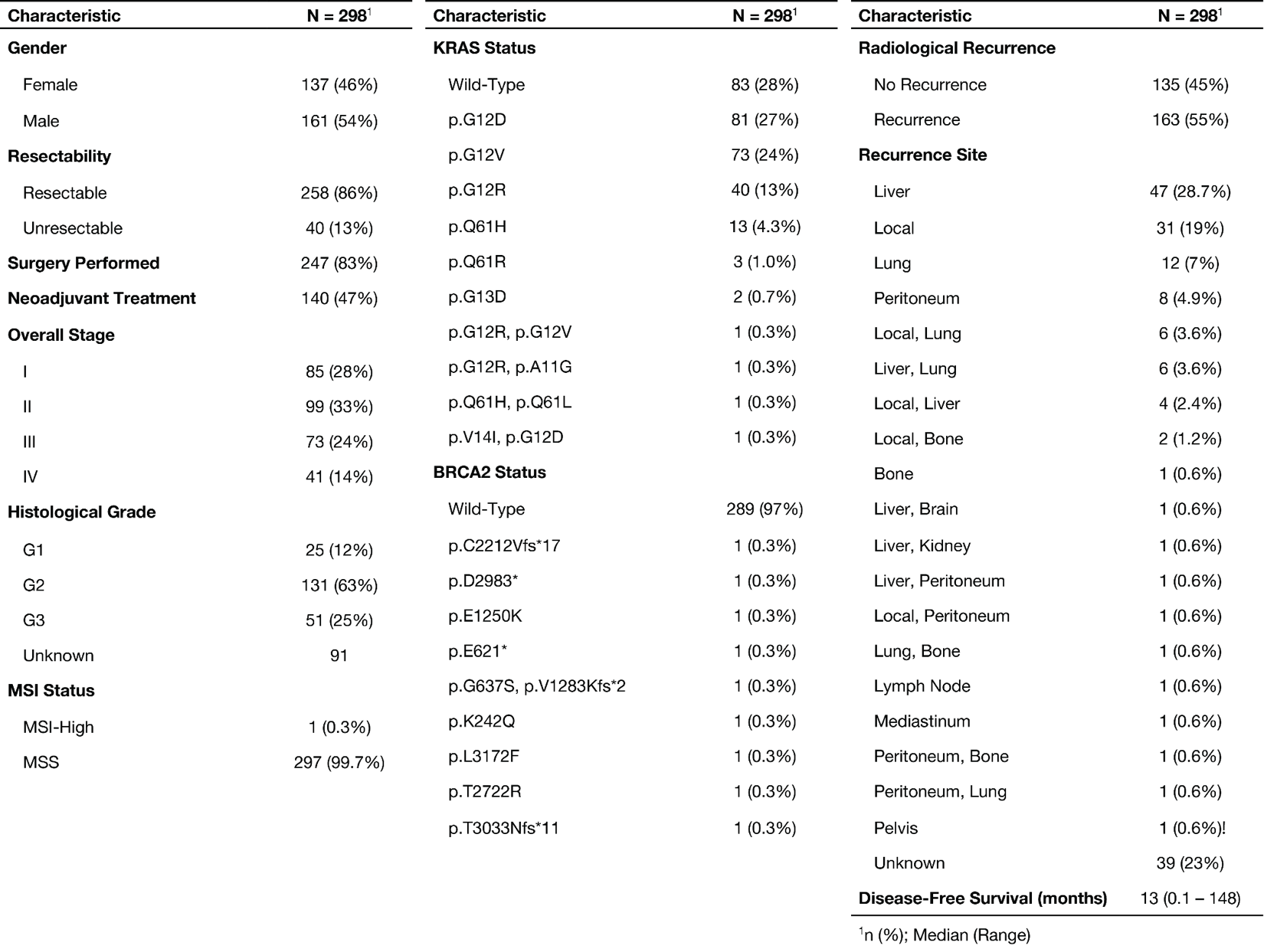


**B**


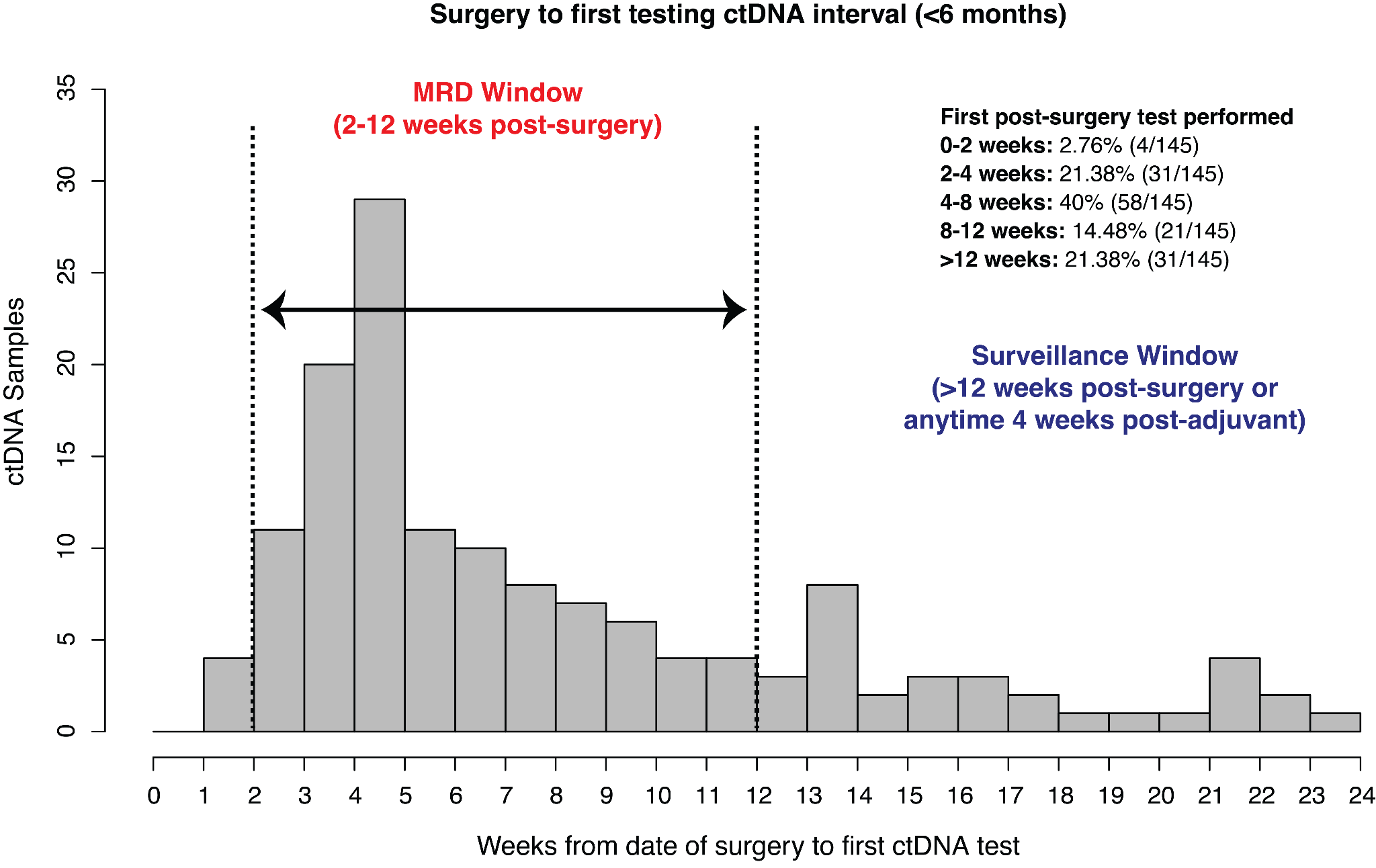


**Supplementary Fig 1: A.** Cohort demographics. **B.** Number of ctDNA samples tested at first post-operative time point (MRD window: 2-12 weeks post-surgery) and during surveillance (>12 weeks up to 24 weeks only).


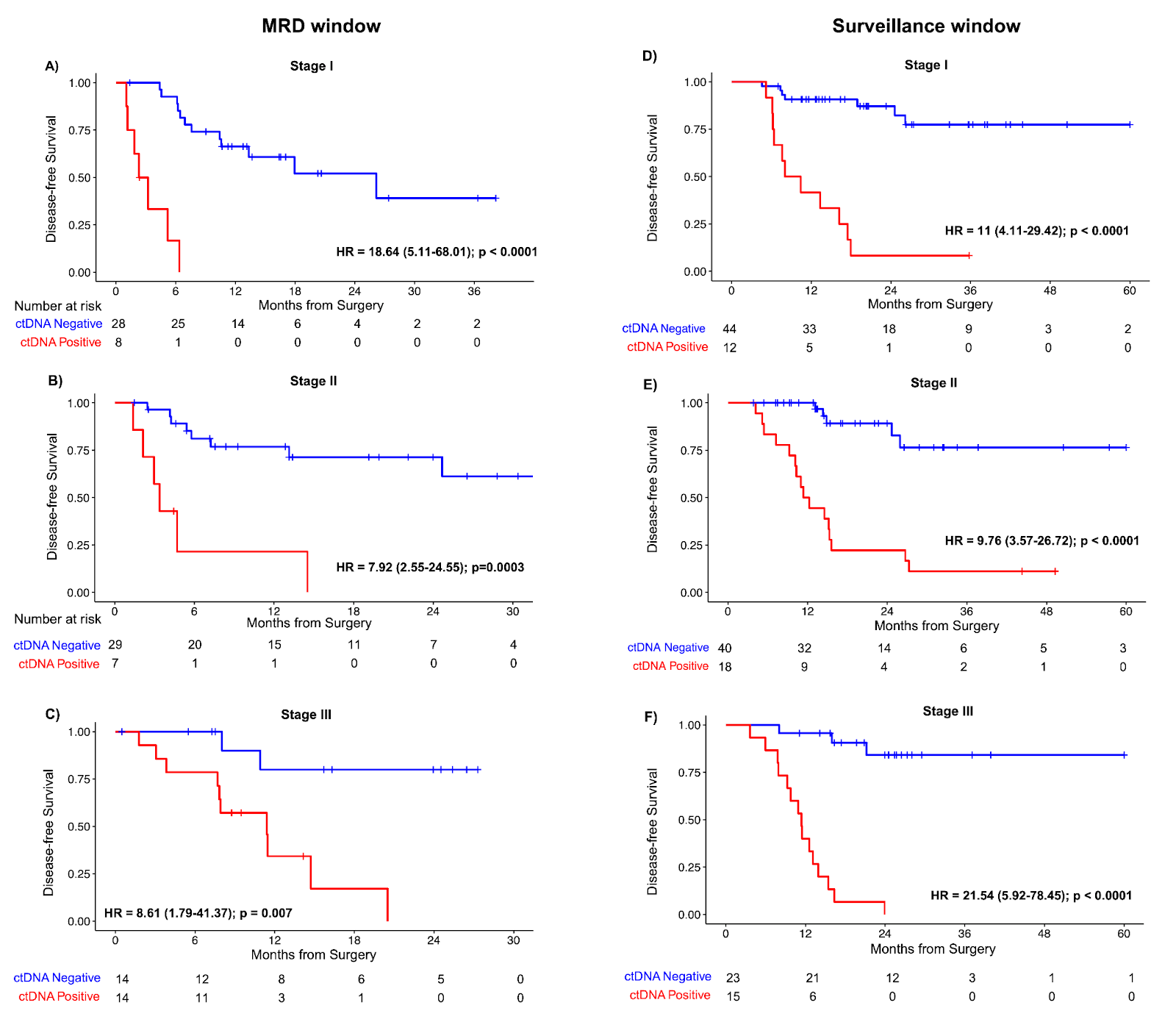


**Supplementary Fig 2: ctDNA-based testing during MRD and surveillance window is predictive of survival outcomes in postsurgical patients with pancreatic cancer. A-C.** Kaplan–Meier estimates for DFS stratified by ctDNA-negative and ctDNA-positive status from 2-16 weeks after surgery, across stage I-III. **D-F.** Kaplan–Meier estimates for DFS stratified by ctDNA-negative and ctDNA-positive status from >12 weeks after surgery. HRs and 95% CIs were calculated using the Cox proportional hazard model. P values were calculated using the two-sided log-rank test.
